# The LAC Score Indicates Significant Fibrosis in Patients With Chronic Drug-Induced Liver Injury: A Large Biopsy-Based Study

**DOI:** 10.3389/fphar.2021.734090

**Published:** 2021-08-18

**Authors:** Zhong-Bin Li, Dan-Dan Chen, Qing-Juan He, Le Li, Guangde Zhou, Yi-Ming Fu, Ya Deng, Xiao-Xia Niu, Fang Chu, Xiao-Pan Gao, Zhengsheng Zou, Guofeng Chen, Dong Ji

**Affiliations:** ^1^ Senior Department of Hepatology, Fifth Medical Center of Chinese PLA General Hospital, Beijing, China; ^2^ Department II of Hepatology, The Second People’s Hospital of Jingzhou City, Jingzhou, China; ^3^ Department II of Gastroenterology, The Eighth People’s Hospital of Qingdao, QingDao, China; ^4^ Senior Department of Infectious Diseases, Fifth Medical Center of Chinese PLA General Hospital, Beijing, China; ^5^ Department of Pathology, Third People’s Hospital of Shenzhen, Shenzhen, China; ^6^ The Second School of Clinical Medicine, Southern Medical University, Guangzhou, China; ^7^ Department of Outpatients, Fifth Medical Center of Chinese PLA General Hospital, Beijing, China; ^8^ Department of Clinical Laboratory, Second Medical Center of Chinese PLA General Hospital, Beijing, China; ^9^ Peking University 302 Clinical Medical School, Beijing, China; ^10^ Chinese PLA 307 Medical College of Anhui Medical University, Beijing, China

**Keywords:** drug-induced liver injury, noninvasive algorithm, fibrosis, liver stiffness measurement, liver biopsy

## Abstract

Currently, there are no satisfactory noninvasive methods for the diagnosis of fibrosis in patients with chronic drug-induced liver injury (DILI). Our goal was to develop an algorithm to improve the diagnostic accuracy of significant fibrosis in this population. In the present study, we retrospectively investigated the biochemical and pathological characteristics of consecutive patients with biopsy-proven chronic DILI, who presented at our hospital from January 2013 to December 2017. A noninvasive algorithm was developed by using multivariate logistic regression, receiver operating characteristic (ROC) curves, and decision curve analysis (DCA) to diagnose significant fibrosis in the training cohort, and the algorithm was subsequently validated in the validation cohort. Totally, 1,130 patients were enrolled and randomly assigned into a training cohort (n = 848) and a validation cohort (n = 282). Based on the multivariate analysis, LSM, CHE, and APRI were independently associated with significant fibrosis. A novel algorithm, LAC, was identified with the AUROC of 0.81, which was significantly higher than LSM (AUROC 0.78), CHE (AUROC 0.73), and APRI (AUROC 0.68), alone. The best cutoff value of LAC in the training cohort was 5.4. When the LAC score was used to diagnose advanced fibrosis and cirrhosis stages, the optimal cutoff values were 6.2 and 6.7, respectively, and the AUROC values were 0.84 and 0.90 in the training cohort and 0.81 and 0.83 in the validation cohort. This study proved that the LAC score can contribute to the accurate assessment of high-risk disease progression and the establishment of optimal treatment strategies for patients with chronic DILI.

## Introduction

Drug-induced liver injury (DILI) refers to liver injury caused by a variety of drugs, herbs, and dietary supplements with liver toxicity under the reasonable exclusion of other causes (Kullak-Ublick et al., 2017; Navarro et al., 2017; European Association for Study of Liver (EASL), 2019). DILI is becoming increasingly concerning as a medical, scientific, and public health problem. The majority of patients with symptomatic acute DILI can completely recover after withdrawing the offending agents, but a small proportion of patients can develop chronic liver disease (Chen et al., 2015; Weaver et al., 2020; Wang et al., 2021). Therefore, before liver injury fully returns to normal, patients with DILI should undergo liver biochemical examination regularly.

Chronic DILI may lead to worse clinical outcomes, such as persistent hepatocyte inflammation, the hepatic fibrosis progression, or an incidence of hepatocellular carcinoma (Kleiner, 2017). Significant fibrosis is the sign of the progression of chronic liver disease. Therefore, the diagnosis of significant fibrosis is of fundamental significance for subsequent clinical treatment decision making. Liver biopsy is a special examination to diagnose substantial liver injury, so as to provide assistant information and help with the accurate diagnosis and prognosis of liver disease (Kleiner et al., 2014; Andrade et al., 2019). However, patients often refuse to receive a biopsy to assess the severity of the disease, which can delay treatment. In addition, liver biopsy is also hindered by a sampling bias (Regev et al., 2002; Martinez et al., 2011) With the application of noninvasive liver fibrosis markers in clinical practice in recent years, the monitoring of chronic liver disease is increasingly less dependent on liver biopsy.

At present, a large amount of research has focused on noninvasive methods to reduce the limitations of liver biopsy. Liver stiffness measurement (LSM), as one of the most promising noninvasive method for the evaluation of hepatic fibrosis, has been widely applied in clinical practice (Boursier et al., 2012; Ji et al., 2021; Li et al., 2021). Several algorithms (combining LSM and serum biomarkers) have been proposed to further improve the diagnostic accuracy of significant fibrosis in patients with hepatitis B/C virus infection (Wong et al., 2014; EASL, 2015; Ji et al., 2021); however, this combined method is used relatively rarely in patients with chronic DILI, thus requiring further study.

Due to the lack of a specific and practical noninvasive method, it is necessary to develop an algorithm that is based on liver biopsy data for the accurate diagnosis of significant fibrosis in patients with chronic drug-induced liver injury. In this study, we aimed to establish and validate a new algorithm (combining LSM and serum biomarkers) to improve the diagnostic accuracy of significant fibrosis.

## Materials and Methods

### Patients

This retrospective study cohort consisted of consecutive patients who received treatment at Fifth Medical Center of Chinese PLA General Hospital, Beijing, China. We enrolled patients who met all of the following inclusion criteria: (1) patients who presented to our hospital from January 2013 to December 2017; (2) patients who were over 18 years old; (3) patients who were diagnosed with chronic DILI, which was defined as DILI with an acute presentation in which there is evidence of persistent liver injury >1 year after its onset (Sarges et al., 2016). and (4) patients who agreed to liver biopsy. Among the patients, we excluded patients who met one of the following exclusion criteria: (1) patients who were complicated with viral hepatitis; (2) patients with DILI combined with other liver diseases, including autoimmune liver disease, alcoholic liver disease, and nonalcoholic fatty liver disease; (3) patients who had any cancers; and (4) patients with a poor quality of biopsy specimens or a high degree of missing data. Clinical laboratory tests, including liver biochemistry and blood cell counts, were performed in local laboratories (in accordance with standard procedures), and liver biopsy results were retrospectively collected within 7 days.

### Randomization

Each enrolled patient was allocated a random number between 0 and 1 (seed = 66) by use of R software (version 4.0.2, https://www.r-project.org) at first step; then they were sorted by the generated random number in ascending order; afterwards, the first three quarters of patients were assigned to a training cohort and rest patients were assigned to a validation cohort (3:1 ratio).

### Liver Stiffness Measurements and Noninvasive Fibrosis Approaches

LSM were performed by experienced operators by using transient elastography (FibroScan^®^; Echosens, Paris, France), based on previously described standard procedures (Ji et al., 2014; EASL, 2015). Only LSM values with 10 valid measurements, success rates ≥60%, and interquartile ranges (IQR)/median ≤30% were considered to be reliable. The aspartate aminotransferase-to-platelet ratio index (APRI) and fibrosis index based on four factors (FIB-4) were calculated for all of the patients by using clinical laboratory data based on the following formulas: APRI = ([AST/ULN]/PLT) × 100 and FIB-4 = (age × AST)/(PLT × ALT^0.5^). (AST ULN = 40 U/L).

### Histological Evaluations

The liver histology of the liver biopsy was independently evaluated by two pathologists. Inconsistencies were further examined by a third senior pathologist. None of the pathologists were aware of the treatments, biopsy sequences, or clinical information. Biopsies with core lengths< 0.5 cm or portal tract numbers <5 were excluded from the final analysis. Necroinflammatory activity was assessed according to the Ishak modified histology activity index (HAI) grading system, which comprises portal inflammation (0–4), interface hepatitis (0–4), focal lytic necrosis and apoptosis (0–4), and confluent necrosis (0–6). Fibrosis stages were assessed with the Ishak fibrosis score, ranging from 0 (no fibrosis) to 6 (cirrhosis).

### Outcomes

The primary outcome was to build a noninvasive fibrosis algorithm to improve the diagnostic accuracy for significant fibrosis (Ishak fibrosis score = 3 points) in chronic DILI patients. The prespecified secondary outcome was to use this algorithm to diagnose advanced fibrosis (Ishak fibrosis score = 4 points) or cirrhosis (Ishak fibrosis score = 5 or 6 points), compared with other noninvasive fibrosis approaches or biomarkers.

### Statistical Analysis

The categorical variables were compared by using a chi-squared test or a Fisher’s exact test. The continuous variables were compared by using an unpaired 2-tailed *t*-test (on normally distributed data) or a Mann-Whitney *U*-test (on skewed distribution data). Univariate analyses were performed to identify variables that were significantly different between patients with and without significant fibrosis. According to the calculated OR and 95% confidence interval (CI), a multivariable logistic regression was further performed to select and eliminate variables. The diagnostic performance of the noninvasive tests was analyzed by using a receiver operating characteristic (ROC) curve analysis that utilized the binomial accuracy method. The ROC area (AUROC) of the different diagnostic methods was compared by using the DeLong method. Sensitivity, specificity, positive predictive value (PPV), negative predictive value (NPV), Youden index, and accuracy were calculated to evaluate the value of the detailed diagnosis. Calibration curves were created to assess the accuracy of the algorithm. A decision curve analysis (DCA) was performed to evaluate the net benefit. A *p*-value < 0.05 was considered to be significant for all of the statistical tests. All of the statistical analyses were performed by using R software and MedCalc Software (version 19.0.4, Ostend, Belgium).

## Results

### Baseline Characteristics

**FIGURE 1:**
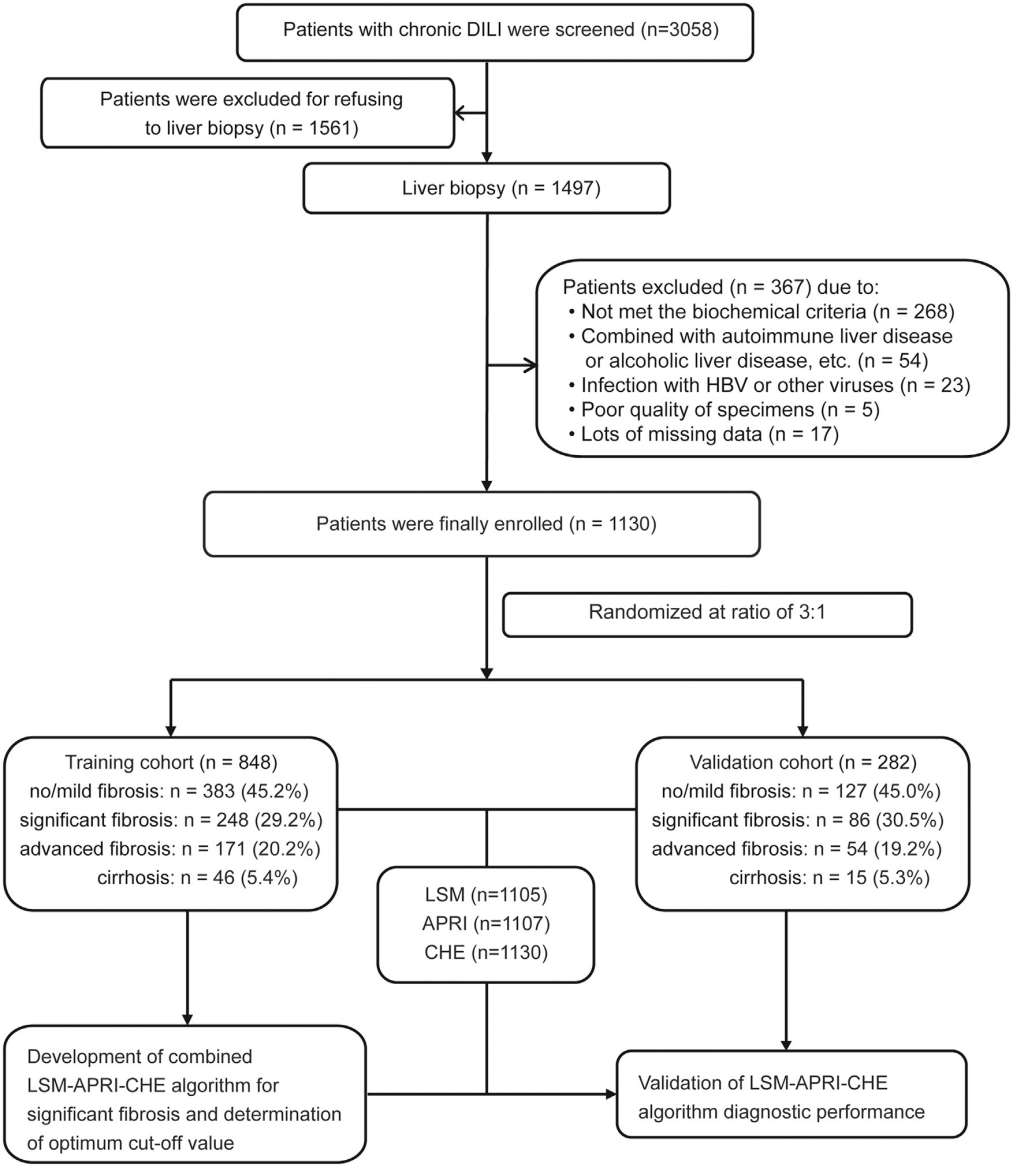
Flow chart of study population.

A total of 3,058 patients with chronic DILI were screened from the Fifth Medical Center of Chinese PLA General Hospital. Of these patients, 1,928 patients were excluded for the reasons listed in Figure 1. The remaining 1,130 patients were included in further analyses and were randomly assigned into a training cohort (n = 848) and into a validation cohort (n = 282) at a ratio of 3:1. The Supplementary Table S1 shows the characteristics of the patients in the training and verification groups. The distribution of sex, age, ALT, AST, ALP, GGT, TBIL, CHE, course of the disease, fibrosis stage, and inflammation grade in the two groups were well matched. Noninvasive fibrosis methods (LSM, APRI, and FIB-4) did not significantly differ between the training and validation cohorts. The characteristics of the patients with or without significant fibrosis in the training cohort are shown in Table 1.

**TABLE 1:** Characteristics of Patients with or without Significant Fibrosis in Training Cohort.

	No/mild fibrosis cohort (*N* = 383)	Significant fibrosis cohort (*N* = 465)	*p* Value
Male sex, *n* (%)	166 (43.3)	156 (33.5)	0.004
Age, years	41.3 (12.4)	45.0 (13.4)	<0.001
BMI, kg/m^2^	23.4 (21.2.25.9)	23.6 (21.5.25.6)	0.864
Course of disease, months	15.0 (1.8)	15.0 (1.7)	0.692
ALT, U/L	59.0 (29.0.112.0)	67.0 (33.0.136.0)	0.035
AST, U/L	42.0 (27.0.77.0)	63.0 (38.0.118.0)	<0.001
ALP, U/L	85.0 (57.0.120.5)	133.0 (78.9,187.0)	<0.001
GGT, U/L	25.0 (19.0.39.8)	41.0 (32.2.67.6)	0.028
TBIL, µmol/L	15.4 (12.0.19.0)	18.4 (13.5.22.3)	0.174
PLT, ×10^9^/L	208.0 (169.5,244.5)	180.0 (134.0.223.0)	<0.001
RUCAM scale, points	8.0 (5.9.10.0)	8.0 (6.0.9.9)	0.978
Length of specimen, mm	11.9 (10.0.18.1)	12.0 (10.1.18.0)	0.999
Histology activity index, *n* (%)			<0.001
1–4 (minor inflammation)	263 (68.7%)	60 (12.9%)	
5–8 (moderate inflammation)	99 (25.8%)	225 (48.4%)	
9–12 (advanced inflammation)	21 (5.5%)	168 (36.1%)	
13–18 (Severe inflammation)	0	12 (2.6%)	
Ishak fibrosis score, n (%)			<0.001
0–2 (no/mild fibrosis)	383 (100.0)	0 (0.0)	
3 (significant fibrosis)	0 (0.0)	248 (53.3)	
4 (advanced fibrosis)	0 (0.0)	171 (36.8)	
5–6 (cirrhosis)	0 (0.0)	46 (9.9)	
LSM, kPa	6.0 (4.7.8.4)	11.9 (7.1.19.7)	<0.001
APRI	0.56 (0.32.1.04)	1.02 (0.56.1.85)	<0.001
FIB-4	1.15 (0.73.2.08)	2.21 (1.17.3.94)	<0.001
CHE, Log_10_ U/L	3.84 (3.77.3.90)	3.73 (3.64.3.83)	<0.001

BMI, body mass index; ALT, alanine aminotransferase; AST, aspartate aminotransferase; ALP, alkaline phosphatase; GGT, gamma glutamyl transpeptidase; TBIL, total bilirubin; PLT, platelet; RUCAM scale, Roussel Uclaf Causality Assessment Method scale; LSM, liver stiffness measurement; APRI, aspartate aminotransferase-to-platelet ratio index; FIB-4, fibrosis index based on four factor; CHE, cholinesterase.

### Development of An Algorithm for Improving Diagnostic Accuracy for Significant Fibrosis

All of the variables that were used in this analysis were based on the data obtained at the time that the liver biopsies were performed. The results of the univariate logistic analysis are presented in Table 2. A multivariate analysis was performed to select fibrosis-related variables. The results that were reported as odds ratio (95% CI), LSM (1.151 [1.120–1.194]), CHE (0.027 [0.007–0.103]), and APRI (1.262 [1.083–1.479]) were independently associated with significant fibrosis (Table 2).

**TABLE 2:** Univariate and multivariate analyses for factors associating with significant fibrosis in chronic DILI patients.

Factors	Univariate		Multivariate	
	Or (95% CI)	*p* value	Or (95% CI)	*p* value
Male sex, *n* (%)	1.420 (1.071–1.882)	0.054	—	—
Age, years	1.022 (1.013–1.032)	<0.001	1.011 (0.994–1.025)	0.461
BMI, kg/m^2^	1.001 (0.987–1.019)	0.567	—	—
ALT, U/L	1.004 (0.982–1.013)	0.021	1.096 (0.995–1.100)	0.371
AST, U/L	1.017 (1.003–1.035)	<0.001	1.015 (1.009–1.037)	0.094
ALP, U/L	1.011 (1.002–1.023)	<0.001	0.984 (0.965–1.012)	0.377
GGT, U/L	1.031 (1.012–1.056)	0.043	1.013 (0.959–1.050)	0.672
TBIL, µmol/L	1.014 (0.979–1.035)	0.472	—	—
PLT, ×10^9^/L	0.986 (0.981–1.009)	<0.001	1.099 (0.995–1.105)	0.173
LSM, kPa	1.201 (1.161–1.237)	<0.001	1.151 (1.120–1.194)	<0.001
APRI	1.798 (1.445–2.003)	<0.001	1.262 (1.083–1.479)	0.004
FIB-4	1.422 (1.303–1.569)	<0.001	1.041 (0.902–1.221)	0.258
CHE, Log_10_ U/L	0.002 (0.001–0.006)	<0.001	0.027 (0.007–0.103)	<0.001

BMI, body mass index; ALT, alanine aminotransferase; AST, aspartate aminotransferase; ALP, alkaline phosphatase; GGT, gamma glutamyl transpeptidase; TBIL, total bilirubin; PLT, platelet; LSM, liver stiffness measurement; APRI, aspartate aminotransferase-to-platelet ratio index; FIB-4, fibrosis index based on four factors; CHE, cholinesterase; OR, odds ratio; CI, confidence interval.

The binary logistic regression was performed to further create an equation based on the combination of noninvasive tests in the training cohort. The LSM-APRI-CHE (LAC) score was identified with the highest AUROC for the indication of significant fibrosis among all of the tests. The equation was LAC = e^c^/(1 + e^c^) × 10, c = 12.109 + 0.143 × LSM (kPa) + 0.23 × APRI − 3.601 × CHE (Log_10_ U/L).

### Correlation Between LAC and Fibrosis Stages

**FIGURE 2:**
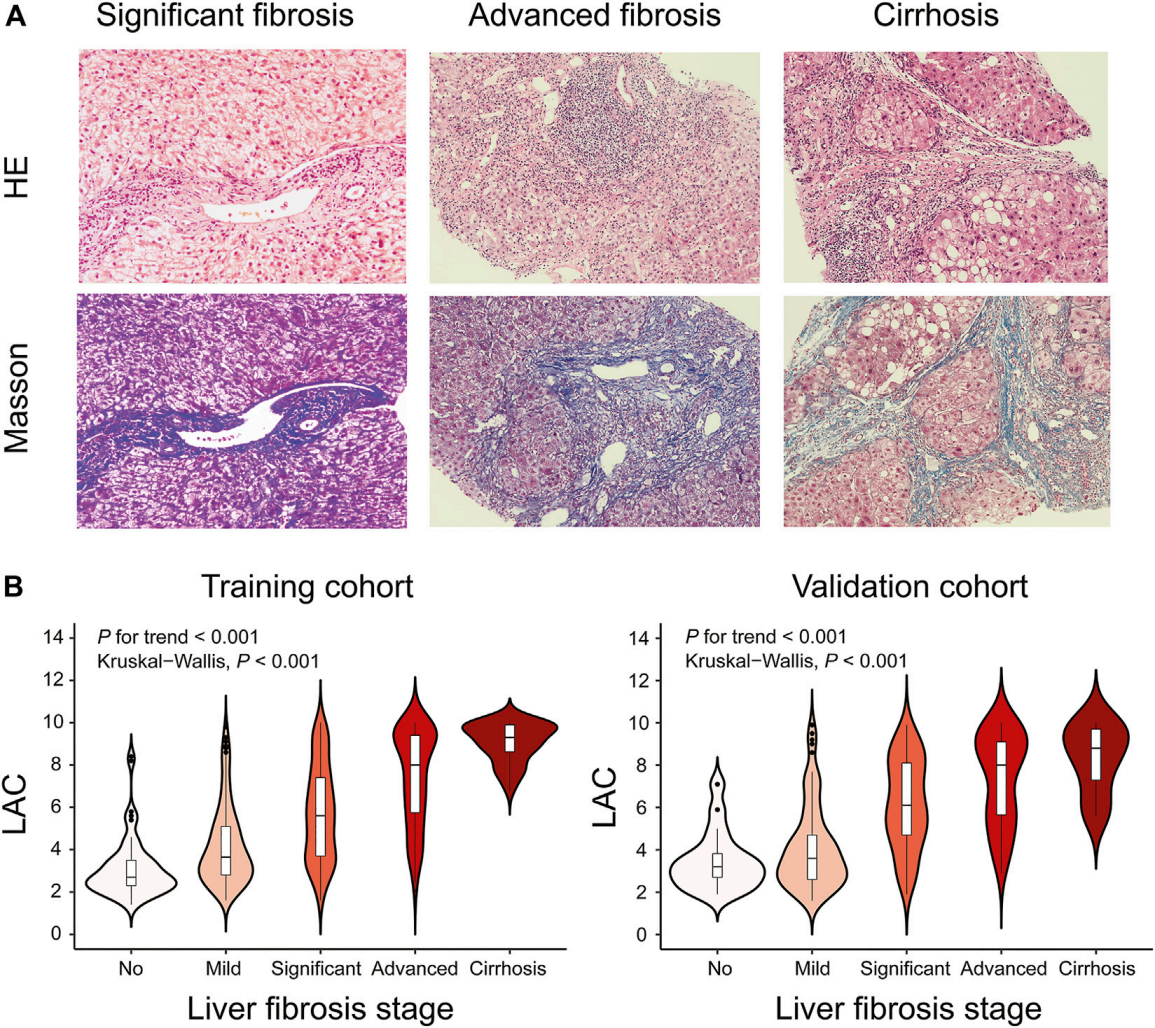
Representative Histological Assessments and Correlation between LAC and Different Fibrosis Stages. Black short dashes in the middle are the median values, and the bars represent the interquartile ranges. The whiskers show 95% CI, whereas the shape of the violin displays the frequencies of the values. **(A)** Hematoxylin-eosin (HE) and Masson trichrome staining of DILI patients with different fibrosis stages (original magnification ×200). For significant fibrosis, regional balloon-like changes, scattered focal necrosis, and apoptotic bodies in the hepatocytes were observed, and the portal area was enlarged with proliferation of the fibrous tissue. For advanced fibrosis, hepatocyte fusion, focal necrosis, and bridging necrosis were easily observable, and the portal area was enlarged with the formation of the fibrous septum. For cirrhosis, regional balloon-like changes in the hepatocytes were observed with more spotted focal necrosis and occasional fusion focal necrosis. The hepatic lobule structure was disordered with the formation of pseudolobule. **(B)** Violin plots showing median LAC values according to the fibrosis stages in the training cohort or validation cohort. Liver fibrosis stages (no, Ishak fibrosis score = 0; mild, Ishak fibrosis score = 1 or two; significant, Ishak fibrosis score = 3; advanced, Ishak fibrosis score = 4; cirrhosis, Ishak fibrosis score = 5 or 6).

Representative liver biopsies of significant, advanced fibrosis and cirrhosis from three patients are shown in Figure 2A. The median (IQR) LAC values from no fibrosis to cirrhosis in the training cohort were 2.7 (2.3–3.5), 3.7 (2.8–5.1), 5.6 (3.7–7.4), 8.0 (5.8–9.4), and 9.3 (8.6–9.9) (Kruskal-Wallis *p* < 0.001), respectively. The corresponding values in the validation cohort were 3.2 (2.7–3.8), 3.6 (2.6–4.7), 6.1 (4.7–8.1), 8.0 (5.7–9.1), and 8.8 (7.3–9.7) (Kruskal-Wallis *p* < 0.001), respectively. The trend test showed that LAC was stepwise increased across the different fibrosis stages (*p* for trend <0.001) in both the training and validation cohorts (Figure 2B).

### Diagnostic Accuracy of LAC

**FIGURE 3:**
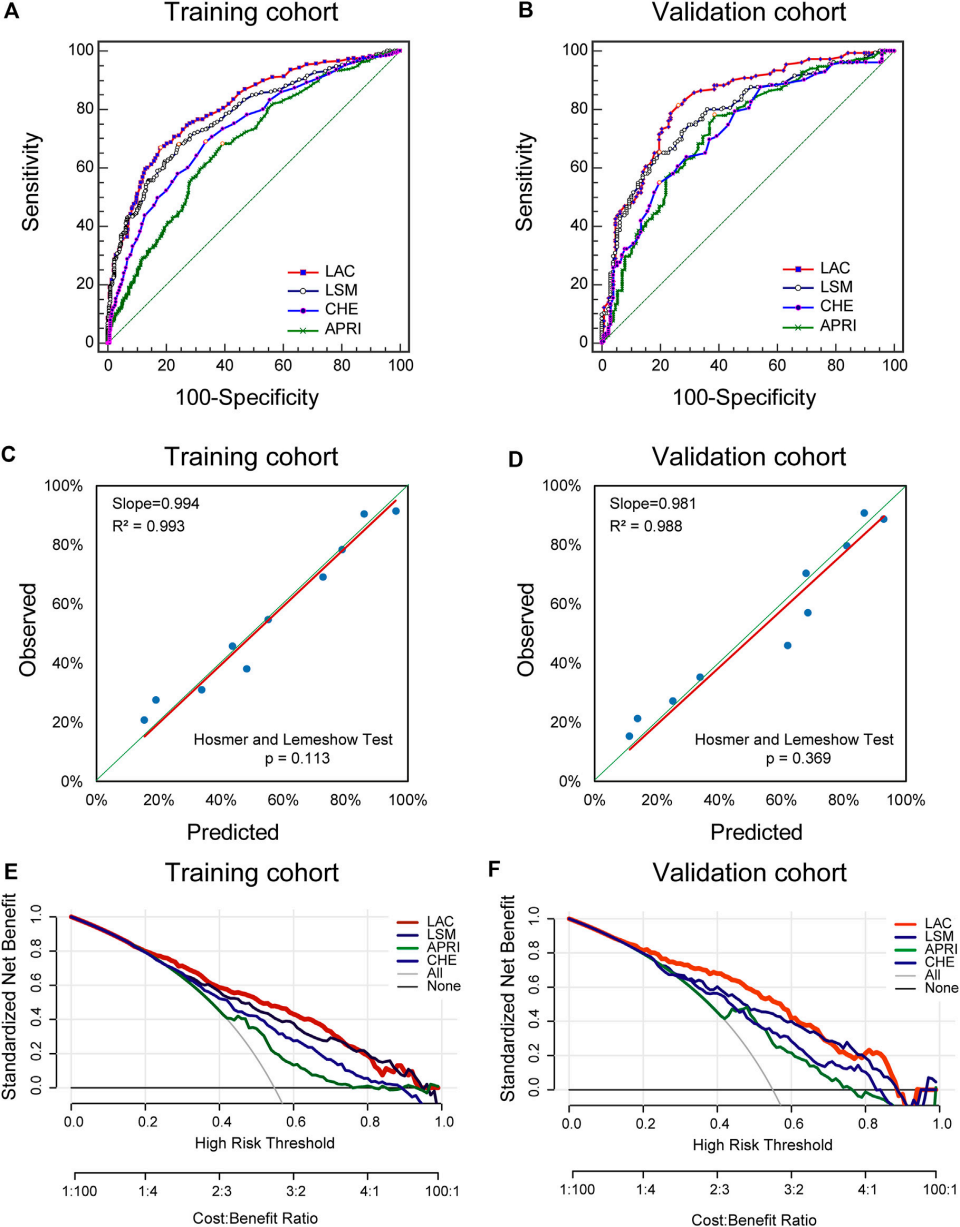
Diagnostic Performance of Significant Fibrosis in the Training and Validation cohorts. ROC, receiver operating characteristics; LSM, liver stiffness measurement; APRI, aspartate aminotransferase-to-platelet ratio index; CHE, cholinesterase. The ROC curves of LAC, LSM, APRI, and CHE in indicating the presence of significant fibrosis in the training cohort. **(A)** and validation cohort **(B)**. Validity of the diagnostic performance of the LAC in the training cohort **(C)** and validation cohort **(D)**. The clinical utility of the LAC and its components were evaluated by decision curves in the training cohort **(E)** and the validation cohort **(F)**.

For the indication of significant fibrosis in the training cohort, the LAC score provided the highest AUROC of 0.81 (95% CI: 0.78–0.83) among all of the methods (Figure 3A). LAC had a better diagnostic performance than LSM (AUROC: 0.78, 95% CI: 0.75–0.80, *p* < 0.05), CHE (AUROC: 0.73, 95% CI: 0.70–0.76, *p* < 0.05), and APRI (AUROC: 0.68, 95% CI: 0.65–0.71, *p* < 0.05). We investigated the optimum cutoff value of LAC for diagnosis by maximizing the sum of sensitivity and specificity, and the best cutoff value of LAC in the training cohort was 5.4. Using the cutoff value, these analyses were repeated in the validation cohort to confirm the validity of the results (Figure 3B).

The resulting LAC score was internally validated by using graphical calibration plots, which showed good agreement regarding the presence of significant fibrosis between the LAC and histopathologic confirmation on the liver biopsy specimens (slopes [*R* ^2^] were 0.994 [0.993]) (Figure 3C). In the validation cohort, there was also a good calibration curve of LAC for the diagnostic accuracy of significant fibrosis (slopes [*R* ^2^] were 0.981 [0.988]) (Figure 3D).

In addition, DCA was used to evaluate the clinical utility of LAC by quantifying the probability of a net gain at a threshold of 0.0–1.0. The further that the decision curve is from the two extremum curves, the net clinical benefit of the algorithm will be higher. In the training and validation queues, the decision curve shows that LAC has a higher net return than any returns of LSM, APRI, or CHE alone (Figures 3E,F). These results suggest that LAC can accurately diagnose significant fibrosis, thus reducing the need for liver biopsy for the assessment of fibrosis severity in patients with chronic DILI.

### Using LAC to Diagnose Advanced Fibrosis and Cirrhosis Stages

Using the LAC score to diagnose advanced fibrosis stages, the optimal cutoff value of the LAC was determined to be 6.2. The AUROC of LAC was 0.84 (95% CI: 0.81–0.86) in the training cohort and 0.81 (95% CI: 0.76–0.85) in the validation cohort. It was significantly higher than LSM, CHE, and APRI (*p* < 0.05). Using the LAC score to diagnose cirrhosis stages, the optimal cutoff value of the LAC was determined to be 6.7. The AUROC of LAC was 0.90 (95% CI: 0.88–0.92) in the training cohort and 0.83 (95% CI: 0.78–0.87) in the validation cohort. It was significantly higher than the values of CHE and APRI (*p* < 0.05) and showed a similar diagnostic performance to LSM (*p* = 0.465) (Table 3 and Supplementary Figure S1).

**TABLE 3:** Diagnostic performance of LAC, LSM, APRI, and CHE in different fibrosis stages.

Significant fibrosis	AUROC (95%CI)	PPV (%)	NPV (%)	Sens (%)	Spec (%)	Youden index
LAC						
Training	0.81 (0.78–0.83)	82.0	67.0	66.7	82.3	0.49
Validation	0.83 (0.78–0.87)	80.1	76.2	80.6	75.6	0.56
LSM						
Training	0.78 (0.75–0.80)	77.3	66.1	68.0	75.7	0.44
Validation	0.78 (0.73–0.83)	80.2	65.4	65.2	80.3	0.45
CHE						
Training	0.73 (0.70–0.76)	71.4	63.8	66.6	68.8	0.35
Validation	0.73 (0.67–0.78)	77.3	59.3	54.8	80.3	0.35
APRI						
Training	0.68 (0.65–0.71)	67.9	61.2	68.2	60.8	0.29
Validation	0.73 (0.67–0.78)	71.2	69.6	78.1	61.4	0.39
**Advanced fibrosis**						
LAC						
Training	0.84 (0.81–0.86)	52.8	90.9	77.9	76.1	0.54
Validation	0.81 (0.76–0.85)	42.6	92.5	84.1	63.4	0.47
LSM						
Training	0.81 (0.78–0.84)	58.2	88.9	70.1	82.7	0.53
Validation	0.78 (0.73–0.83)	51.1	88.8	69.6	78.4	0.48
CHE						
Training	0.74 (0.71–0.77)	38.8	89.2	80.2	56.6	0.37
Validation	0.70 (0.65–0.76)	40.0	85.5	63.8	69.0	0.33
APRI						
Training	0.68 (0.65–0.72)	38.6	83.8	63.1	65.5	0.29
Validation	0.69 (0.64–0.75)	36.4	89.8	81.2	54.0	0.35
**Cirrhosis**						
LAC						
Training	0.90 (0.88–0.92)	17.1	100.0	100.0	72.2	0.72
Validation	0.83 (0.78–0.87)	11.5	100.0	100.0	56.9	0.57
LSM						
Training	0.91 (0.75–0.80)	21.9	99.5	93.5	80.9	0.74
Validation	0.85 (0.80–0.89)	15.6	99.5	93.3	71.5	0.65
CHE						
Training	0.75 (0.72–0.78)	13.6	97.7	69.6	74.7	0.44
Validation	0.68 (0.63–0.74)	10.6	97.3	66.7	68.5	0.35
APRI						
Training	0.70 (0.66–0.73)	9.2	98.2	82.6	53.1	0.41
Validation	0.66 (0.60–0.71)	9.6	98.6	86.7	53.9	0.39

LSM, liver stiffness measurement; APRI, aspartate aminotransferase-to-platelet ratio index; CHE, cholinesterase; AUROC, area under the ROC curve; CI, confidence interval; PPV positive predictive value; NPV, negative predictive value; Sens, sensitivity; Spec, specificity.

## Discussion

Most patients who experience DILI will clinically recover, and their liver function will return to normal. However, chronic onset and progression to cirrhosis were observed during the follow-up of liver injury, and continuously elevated transaminases may indicate chronic outcomes practice (Fontana et al., 2014; Raschi and De Ponti, 2017). We retrospectively analyzed the clinical data of 1,130 patients with biopsy-proven chronic DILI and found that LSM, APRI, and CHE were related to fibrosis stage. Subsequently, we set up a new noninvasive algorithm, named as LAC, with the sufficient performance in indicating significant fibrosis, which could predict the risk of disease progression and assist to establish the individualized and precise therapeutic strategy for patients with chronic DILI.

For the diagnosis of chronic DILI, the cutoff point for chronic conditions is still a hot topic of debate (Sarges et al., 2016). In a study of 598 patients prospectively confirmed cases of DILI by the DILIN group in the United States, 18.9% of the patients showed signs of persistent liver injury, and chronic DILI was defined as an elevated liver test 6 months after inclusion with histological or radiological evidence indicating persistent liver injury (Dear, 2018). The DILI Registry in Spain analyzed the time it took for liver function to return to normal in 298 DILI patients to determine the optimal cutoff point for chronic DILI, with the results suggesting that 1 year is the optimal cutoff point for defining chronic DILI or prolonged recovery (Medina-Caliz et al., 2016). The EASL guidelines also recommend that 1 year is the best cutoff point for a chronic condition (EASL, 2019). Therefore, we enrolled patients based on these criteria, and the results showed that the average course of disease was 15.0 months, which is consistent with the Spanish cohort results.

LSM is an ultrasound-based elastography technique that is used in clinical practice, and both APRI and FIB-4 are also recommended by the WHO guidelines for clinical fibrosis assessment. Therefore, we used LSM, APRI, and FIB-4 to assess liver fibrosis due to the convenient, noninvasive, and quantitative features. In the present study, LSM (AUROC: 0.78, 95% CI: 0.75–0.80) also showed good diagnostic efficiency for significant fibrosis. However, APRI and FIB-4 have been reported to have poor diagnostic accuracy in patients with chronic liver disease. As our study showed, FIB-4 (OR: 1.04, 95% CI: 0.90–1.22, *p* = 0.258) was not independently associated with significant fibrosis in patients with chronic DILI, and the AUROC of APRI (0.68, 95% CI: 0.65–0.71) was lower than that of other noninvasive approaches in our study. Notably, our study found that CHE (OR: 0.027, 95% CI: 0.007–0.103, *p* < 0.001) was independently associated with significant fibrosis in chronic DILI patients, and the AUROC (0.73, 95% CI: 0.70–0.76) was higher than the APRI. To date, there are no satisfactory serum biomarkers or detection methods to evaluate fibrosis in patients with chronic DILI (Miao et al., 2010; Chruch and Watkins, 2017), and CHE may be a good candidate indicator. CHE has been used to evaluate liver reserve function in patients with chronic liver disease (Liu et al., 2019), but there is little data to discuss whether it can be used to indicate the degree of liver fibrosis. Theoretically, when the liver parenchyma is injured, a decrease in the normal hepatocyte number and changes in liver architecture will promote the progression of fibrosis and an obvious decline in CHE activity. This mechanism has been supported by many studies (Ramachandran et al., 2014; Lutterkort et al., 2017), which implied that CHE activity in chronic liver disease was significantly and negatively correlated with histopathological necrosis, inflammation grade, and fibrosis stage of the liver, and there were also significant differences between the different pathological grades and stages. In addition, from the laboratory’s point of view, the CHE detection method is stable and is not susceptible to other factors (such as ALT or TBIL) (Sussman and Silman, 2020). Therefore, CHE may be important in determining the severity and prognosis of chronic liver disease. According to the previous analyses, this new, easy-to-use algorithm known as LAC score has good diagnostic performance for significant fibrosis in patients with chronic DILI.

Some limitations of the study deserved to be mentioned although the sample size is large of this study. Firstly, our study did not include a longitudinal study to assess whether the LAC score can monitor dynamic changes in fibrosis staging, which warrants further study. Secondly, this analysis was based on data from a single center, and a multicenter study (including participants from different regions or races) is needed for further external validation. Thirdly, although LAC has achieved a good diagnostic accuracy, its sensitivity and specificity still need to be improved. Finally, due to the fact that the model is based on clinicopathological data, the assessment of specific markers of liver fibrosis may further improve accuracy.

The following factors were strengths of our study. (1) Novelty - to the best of our knowledge, the combined use of different noninvasive fibrosis approaches or biomarkers (based on divergent mechanisms) has been widely investigated in HBV/HCV patients, but few studies exist concerning chronic DILI patients. LAC is the first algorithm that was developed for the diagnostic accuracy of significant fibrosis for patients with chronic DILI. (2) A strong practicality - LAC is easy to use and has great potential to be widely implemented in clinical practice, particularly in cases where liver biopsy is limited. (3) A high reliability - the sample size of our study was large enough, and the diagnosis of chronic DILI was confirmed by liver histology, which has both universality and representativeness.

## Conclusion

The LAC score shows a better performance than LSM, APRI, or CHE alone in indicating significant fibrosis, thus reducing the need for liver biopsies to assess the severity of liver fibrosis and contributing to the accurate assessment of high-risk disease progression as well as the establishment of optimal treatment strategies for patients with chronic DILI.

## Data Availability

The original contributions presented in the study are included in the article/Supplementary Material, further inquiries can be directed to the corresponding authors.

## Abbreviations

DILI, drug-induced liver injury; HBV, hepatitis B virus; HCV, hepatitis C virus; ULN, upper limit of normal; BMI, body mass index; ALT, alanine aminotransferase; AST, aspartate aminotransferase; TBIL, total bilirubin; ALP, alkaline phosphatase; GGT, gamma glutamyl transpeptidase; PLT, platelet; CHE, cholinesterase; RUCAM, roussel uclaf causality assessment method; LSM, liver stiffness measurement; APRI, aspartate aminotransferase-to-platelet ratio index; FIB-4, fibrosis index based on four factors; IQR, interquartile range; SD, standard deviation; OR, odds ratio; CI, confidence interval; ROC, receiver operating characteristics; AUROC, area under the ROC curve; DCA, decision curve analysis; EASL, Europeans association for study of liver.
