# Supplementary material for: The LAC Score Indicates Significant Fibrosis in Patients With Chronic Drug-Induced Liver Injury: A Large Biopsy-Based Study
Source: Front Pharmacol. 2021 Aug 18;12:734090. doi: 10.3389/fphar.2021.734090 (PMC8416439; doi:10.3389/fphar.2021.734090)
Supplement: Supplementary file 1 [file DataSheet1.docx]

**The LAC score indicates significant fibrosis in patients with chronic drug-induced liver injury: a large biopsy-based study**

| **SUPPLEMENTARY TABLE** Patients Characteristics in Training and Validation cohort. | | | | |
| --- | --- | --- | --- | --- |
|  | **Overall**  **(n=1130)** | **Training cohort**  **(n=848)** | **Validation cohort**  **(n=282)** | ***P* value** |
| Male sex, n (%) | 423(37.4) | 322(38.0) | 101(35.8) | 0.564 |
| Age, years | 43.0(13.0) | 43.3(13.1) | 42.2(12.7) | 0.225 |
| BMI, kg/m^2^ | 23.4(21.3,25.6) | 23.5(21.3,25.7) | 23.4(21.4,25.0) | 0.377 |
| Course of disease, months | 15.0 (1.7) | 15.0 (1.7) | 15.2 (1.7) | 0.116 |
| ALT, U/L | 61.0(31.0,123.8) | 61.0(31.0,126.5) | 62.0(32.0,122.0) | 0.831 |
| AST, U/L | 54.0(32.0,99.0) | 54.0(32.0,99.0) | 54.0(32.0,103.0) | 0.756 |
| ALP, U/L | 128.0(67.3,165.9) | 127.0(66.9,165.0) | 128.0(67.1,160.6) | 0.694 |
| GGT, U/L | 35.0(27.2,57.7) | 35.0(26.8,58.0) | 34.0(27.0,57.8) | 0.599 |
| TBIL, µmol/L | 16.0(12.0,21.2) | 16.8(12.9,21.7) | 15.3(11.8.20.9) | 0.565 |
| PLT, ×10^9^/L | 192.0(146.0,239.0) | 193.0(148.0,236.0) | 188.5(142.3,250.0) | 0.770 |
| RUCAM scale, points | 8.0(6.0,10.0) | 8.0(6.0,10,0) | 8.0(6.0,9.9) | 0.967 |
| Length of specimen, mm | 12.0(9.9,18.1) | 12.0(10.0,18.0) | 12.0(10.0,17.9) | 0.999 |
| Histology activity index, n (%) | | |  | 0.801 |
| 1-4 (minor inflammation)  5-8 (moderate inflammation)  9-12 (advanced inflammation)  13-18 (Severe inflammation) | 432(38.3%)  414(36.6%)  269(23.8%)  15(1.3%) | 323(38.1%)  324(38.2%)  189(22.3%)  12(1.4%) | 109(39.0%)  90(35.6%)  80(24.4%)  3(1.0%) |  |
| Ishak fibrosis score, n (%) |  |  |  | 0.932 |
| 0-2 (no/mild fibrosis)  3 (significant fibrosis)  4 (advanced fibrosis)  5-6 (cirrhosis) | 510(45.1%)  334(29.6%)  225(19.9%)  61(5.4%) | 383(45.2%)  248(29.2%)  171(20.2%)  46(5.4%) | 127(45.0%)  86(30.5%)  54(19.2%)  15(5.3%) |  |
| LSM, KPa | 8.3(5.5,15.0) | 8.2(5.5,15.0) | 8.4(5.5,15.3) | 0.884 |
| APRI | 0.75(0.41,1.38) | 0.78(0.41,1.44) | 0.81(0.41,1.56) | 0.916 |
| FIB-4 | 1.58(0.88,3.00) | 1.60(0.88,2.99) | 1.54(0.84,3.07) | 0.636 |
| CHE, Log_10_ U/L | 3.79(3.69,3.87) | 3.79(3.69,3.87) | 3.78(3.68,3.87) | 0.481 |

BMI, body mass index; ALT, alanine aminotransferase; AST, aspartate aminotransferase; ALP, alkaline phosphatase; GGT, gamma glutamyl transpeptidase; TBIL, total bilirubin; PLT, platelet; RUCAM scale, Roussel Uclaf Causality Assessment Method scale; LSM, liver stiffness measurement; APRI, aspartate aminotransferase-to-platelet ratio index; FIB-4, fibrosis index based on four factor; CHE, [cholinesterase](javascript:;).


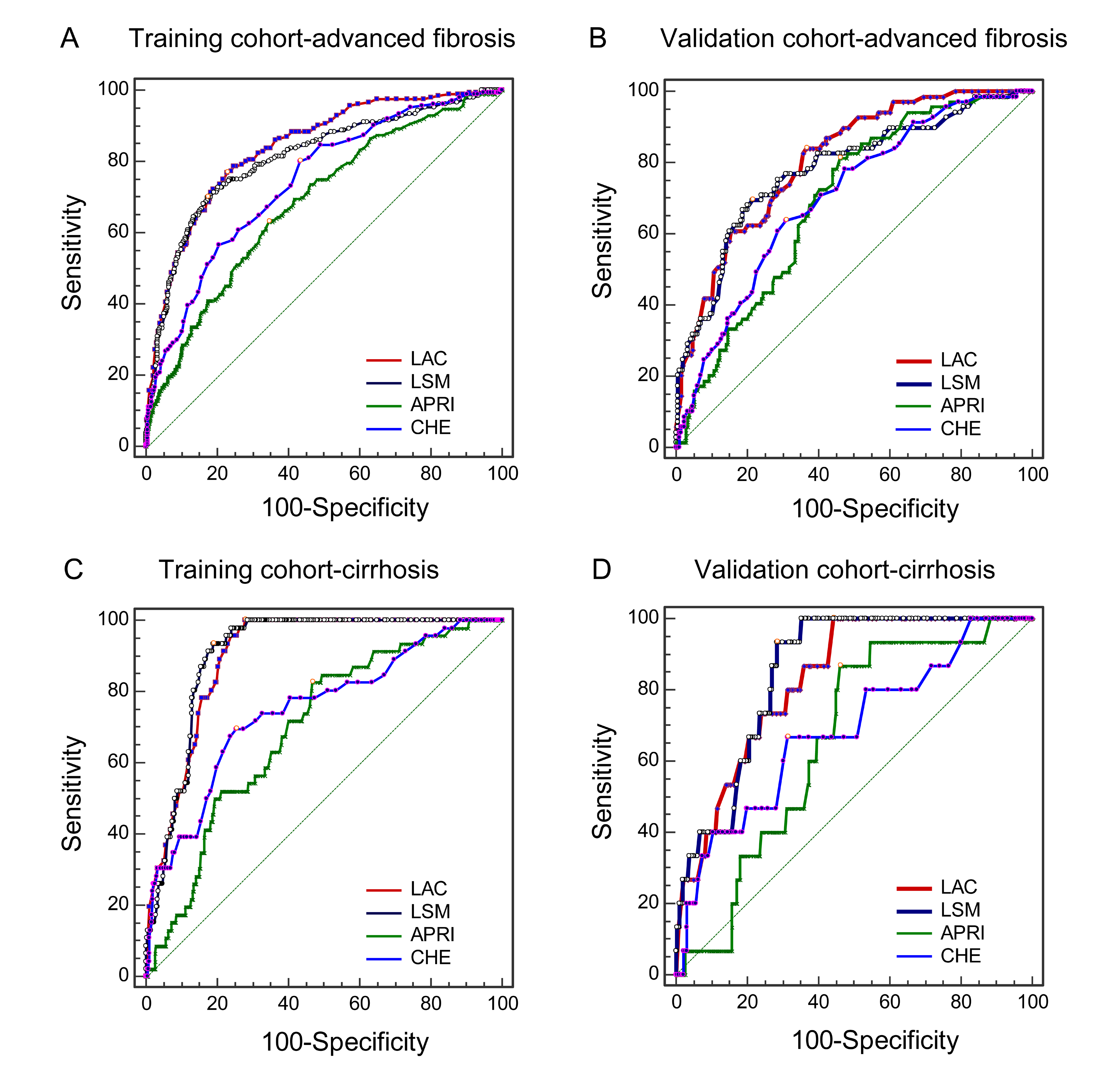


**SUPPLEMENTARY FIGURE.** Diagnostic Performance of Advanced Fibrosis and Cirrhosis Stages in Training and Validation Cohorts. ROC, receiver operating characteristics; LSM, liver stiffness measurement; APRI, aspartate aminotransferase-to-platelet ratio index; CHE, [cholinesterase](javascript:;).
